# Potential New Approaches for Prostate Cancer Management in Resource-Limited Countries in Africa

**DOI:** 10.5334/aogh.3994

**Published:** 2023-02-20

**Authors:** Maarten C. Bosland, Olayiwola B. Shittu, Edet E. Ikpi, Oluyemi Akinloye

**Affiliations:** 1 Department of Pathology and Center for Global Health, University of Illinois at Chicago, Chicago, IL, USA; 2 Department of Surgery, College of Medicine, University of Ibadan, Ibadan, Nigeria; 3 Department of Urology, University of Calabar Teaching Hospital, Calabar, Nigeria; 4 Department of Medical Laboratory Science, Faculty of Basic Medical Sciences, College of Medicine, University of Lagos, Idiaraba, Lagos, Nigeria; 5 Center for Genomics of Non-Communicable Diseases, University of Lagos, Akoka, Lagos, Nigeria

**Keywords:** Prostate cancer, Prostate-specific antigen, Sub-Shaharan Africa, Treatment alternatives, Hormonal therapy, Low resource areas

## Abstract

Prostate cancer is a major male malignancy in many sub-Saharan countries in Africa. Because of resource limitations, screening, early detection, diagnosis, and curative treatments are not available for many men on the subcontinent, and there are even barriers to the treatment of advanced-stage metastatic prostate cancer. We are making the case for new approaches to the detection, diagnosis, and treatment of this malignancy in sub-Saharan Africa and other low-resource regions—approaches that differ from the ones available and used in high-income countries. The development of one-step dipstick-type detection assays of serum prostate-specific antigen (PSA) offers an approach to prostate cancer detection, treatment and monitoring that circumvents issues related to laboratory quality control and is also low-cost. Curative-intent treatments of early-stage prostate cancer are often unavailable in low-resource contexts, and most prostate cancers are not detected in Africa until they are at an advanced stage. Hence, androgen deprivation treatments, including orchiectomy and older low-cost drugs, offer feasible and affordable approaches to prolong survival and sustain a reasonable quality of life. However, clinical trials are needed to identify which of these androgen deprivation treatments are most efficacious and best tolerated to make progress in providing medical care for men with prostate cancer in sub-Saharan Africa and other low- and lower-middle-income areas around the world.

Prostate cancer is reportedly the most common male malignancy in many countries in sub-Saharan Africa (SSA). However, cancer registration is not very reliable in many of these countries as only 6% of the region is currently covered by reasonably accurate death registration systems, and high-quality population-based cancer registration is only present in four countries [1]. Prostate cancer may be overreported [2], cancers in other tissues such as the liver and the colon may be underreported, and cancer incidence rates in Africa are sometimes subject to profound changes over time [3,4]. Nonetheless, prostate cancer surely ranks among the more common male malignancies in Africa and appears to have increased over the past few decades [5]. Despite the high prevalence the detection, diagnosis and treatment of prostate cancer is problematic in SSA for several reasons. The objective of this commentary is to summarize these difficulties and identify some possible approaches to alleviate them.

## Obstacles to Detection, Diagnosis, and Treatment of Prostate Cancer in SSA

### Screening and diagnosis

In the United States and much of Europe, the use of prostate-specific antigen (PSA) for screening remains a matter of great debate [6,7]. As there is little information about prostate cancer screening in SSA and other low- and lower-middle-income countries (LLMICs) [8], the merit of screening in SSA and other low-resource areas remains unclear. In a community-based screening program in Nigeria, 10% of men 50 years and over had a PSA over 4 mg/ml, and most had a PSA greater than 10 mg/ml. 8.5% were biopsied, and 1% had histologically confirmed prostate cancer; no information was provided about treatments and outcomes [9]. In the US and Western Europe, PSA screening resulted in as much as a tenfold rise in prostate cancer incidence but lower rates of negative biopsies, while screening had no effect on all-cause mortality and had mixed results on prostate cancer-specific mortality [7]. Hindering screening is the low awareness of prostate cancer amongst the general population of many SSA countries [10], although there are also areas where awareness is higher [11].

Assays used for testing to detect prostate cancer are highly variable in quality in SSA, as is quality control in clinical laboratories, particularly in private laboratories. PSA testing may be costly as well [12]. Biopsies are the gold standard for diagnosing prostate cancer, but they are often not possible because of the cost and limitations in urology (ultrasound-guided biopsies) and biopsy pathology services [13], particularly in rural areas where 50% of the population live [14]. Hence, biopsy practices likely vary widely in SSA. Digital rectal exams (DRE) are more widely possible, but these do not tend to be well-accepted by patients, are not very sensitive, and not very likely to detect potentially curable early-stage cancers. Consequently, prostate cancer is often not detected until the disease is at an advanced stage, when symptoms such as pain or urinary problems push patients to seek medical care [15,16,17,18,19,20]. Detecting metastatic diseases requires imaging, particularly bone scans, but this is costly, and the required equipment and expertise is not available in many resource-limited settings in SSA or other LLMICs.

### Treatment

Treatment with curative intent is not widely available and is often very costly in SSA [21]. Radiation treatment is limited due to serious shortages of the necessary high-quality equipment and clinical expertise, and the need for repeated treatments requiring travel or temporary relocation, leave from work, and financial resources—all of which may be out of reach for many patients [22]. Surgery (radical prostatectomy) is also costly and requires the urologic surgeon to have and maintain the necessary proficiency to perform this complex operation [23]. Maintaining proficiency requires a sufficiently large volume of eligible patients who can afford this intervention. This is only possible in high-volume hospitals in large cities with sufficient amounts of affluent persons who can afford surgery. In non-urban SSA, a shortage of physicians, particularly urologists and radiation oncologists, further complicates the availability of curative treatments. Consequently, PSA screening is not useful for most men in low-resource settings in LLMICs because biopsy diagnosis and curative treatments cannot be offered, as they are not available or affordable.

Because most prostate cancers in SSA have already progressed to an advanced stage at presentation and are often metastatic, surgical or medical androgen deprivation therapy (ADT) and chemotherapy are the only options for most of those patients. Immuno- and targeted therapies are either not available at all or are far too costly, as one third of the population in SSA live in extreme poverty; in some countries, this number can be as high as 70% [24,25]. And even standard treatments with Lupron (leuprolide) or Zoladex (goserelin) is often not available and/or too costly. Recently developed ADT treatments such as abiraterone and enzalutamide are entirely out of reach financially for most African patients and often not available at all in low- and lower-middle-income countries.

## New Approaches to Detection, Diagnosis, and Treatment of Prostate Cancer in SSA

As summarized above, a host of factors contribute to obstructing the detection, diagnosis, and treatment of prostate cancer: various forms of financial toxicity [8]; limitations with regards to the availability of laboratories, surgeries, radiation treatment, and medical oncological services [21]; lack of prostate cancer awareness and medical literacy; reliance on traditional medicine delaying the pursuit of conventional medical care [26,27,28]. To find more realistic, feasible, and affordable means of providing prostate cancer patients in SSA and other LLMICs with effective medical care, approaches are needed that are different from those used in high- and upper-middle-income countries.

### Screening

In order for screening to be effective, the screening test must find clinically significant diseases earlier than its clinical presentation, and treatment must be available that will alter the natural history of the disease [6]. Recommendations regarding PSA cut-off values and age-specific screenings have not been established for SSA populations, and it is not clear whether PSA screening is feasible and effective in each of the very diverse urban and rural populations of SSA. Thus, research is needed to (1) develop such recommendations for SSA populations, (2) determine whether screening is feasible and effective for all strata of SSA populations, and (3) identify treatments that are feasible and effective in the SSA context, as is discussed later.

### PSA testing and diagnosis

Low-cost alternatives to measuring serum PSA by standard immuno-assays are needed that are not dependent on laboratory quality control limitations. A rapid, one-step PSA test that has the potential to be semi-quantitative has been described using methods similar to those used for rapid COVID-19 antigen tests [29,30]. Although these tests are currently not commercially available to the best of our knowledge, they would greatly improve access to PSA testing in resource-limited and financially restrained settings, particularly when combined with DRE [13]. With the limit of detection of such an assay set at a PSA value high enough to indicate cancer with certainty, the presence of a prostate malignancy could be identified after other causes of PSA elevation are excluded, such as: benign prostatic hyperplasia by DRE and urine flow measurement; prostatitis by clinical symptoms and urine microbiology. An elevated PSA (e.g., ≥30 or 60 ng/ml) could thus be used as a surrogate for a histological biopsy diagnosis [31], and for the initiation of therapy [32]. This would then open up the opportunity for treatment with androgen deprivation therapy, which should drive down PSA levels.

### Treatment

Given that curative treatments are likely to be out of reach for all but a few men with prostate cancer in LLMICs and most patients present with advanced disease, low-cost ADT can reduce symptoms, improve quality of life, and prolong survival in all but some patients with very advanced disease. Orchiectomy is the simplest, one-step approach, albeit often not easily accepted by patients. Alternatively, there are relatively low-cost drugs that can be used, such as flutamide, bicalutamide (Casodex), or cyproterone acetate with low-dose stilbestrol (DES) being the cheapest [33]. These drugs are in pill form and do not require repeated injections, such as with Lupron. However, it is not known which of these androgen deprivation therapy approaches is the most effective with the lowest amount of side effects for different clinical stages of prostate cancer. Small clinical trials comparing them head-to-head at the different stages of prostate cancer would be needed. This could be done with asymptomatic men who have early advanced-stage prostate cancer (e.g. PSA > 60 ng/ml); a downward change of PSA can be easily used as a relevant biomarker endpoint in relatively small and short-term trials. Symptomatic men with high serum PSA could be randomized and monitored for PSA changes and the alleviation of symptoms such as bone pain. This kind of trial may require a larger sample size but clearly reveal beneficial effects on quality of life. Survival, both overall and prostate cancer-specific, would be important to determine. This would require trials with a larger sample size, but ADT methods that are clearly less effective in lowering PSA and/or reducing symptoms in smaller trials could be omitted.

One would think that such clinical trials have already been carried out in low-resource settings. However, this does not appear to be the case when consulting the published literature focusing on LLMICs in PubMed searches; most studies have at best involved non-randomized comparisons of different treatments in small studies [34]. In a retrospective study from China, orchiectomies with or without Casodex or flutamide treatment were compared; the combined treatment was more effective in reducing PSA and increasing prostate cancer-specific survival in men with metastatic disease, but not on non-metastatic patients [35]. This study indicates that further clinical studies of such combined treatments may be promising and that stratifying patients in such studies by clinical stage is important. In a retrospective US study, orchiectomy had the same impact on overall survival as medical ADT, but most likely including low-cost drugs; specifics were not included in the report [36]. Two Nigerian studies compared different techniques of orchiectomy, examining differences in subsequent quality of life but not progression or survival [37,38].

Chemotherapy with docetaxel plus low-dose prednisone for castration-resistant metastatic prostate cancer once ADT has failed may well be out of reach of many low-income patients in SSA, especially given the cost and need for repeated infusions of intravenous docetaxel (a dose of 75 mg/m^2^ given intravenously every 21 days for ten cycles is approved by the US FDA). In addition, increases of average progression-free and overall survival are very modest with this chemotherapy [39]. Palliative treatment is likely more achievable and beneficial for these patients in SSA [40,41].

## Concluding Remarks

New approaches to prostate cancer management in SSA and other LLMICs are needed, approaches that are different from those used in high- and upper-middle-income countries. Although research is needed on the feasibility and effectiveness of screening in low-resource areas, there are simple ways in which testing for and detecting prostate cancer in low-resource contexts is possible. One-step dipstick-type detection assays of serum PSA offer such an approach, which circumvents issues related to quality control and is low-cost. Curative intent treatments of early-stage prostate cancer are often not available in low-resource contexts, and most prostate cancers are not detected in SSA until they are at an advanced stage. Thus, ADT by orchiectomy and older low-cost drugs offer opportunities to prolong survival and reasonable quality of life—methods that are more feasible and affordable in low-resource areas in SSA and other LLMICs. However, clinical trials are needed to identify which of these androgen deprivation treatments are most efficacious and best tolerated. Such trials can use the alleviation of symptoms and the reduction of PSA as endpoints in relatively small and short randomized studies; these are needed to make progress in providing medical care for men with prostate cancer in SSA and other LLMICs around the world. Additionally needed is: the training of more urologist and urologic oncologists, focusing on their ability to perform biopsies and prostate surgery; the improvement of clinical facilities, including those for radiotherapy and laboratory and pathology diagnosis; the development of prostate cancer awareness and education among the general public, as well as the medical community in SSA [42].
